# Identification and In Vivo Characterisation of Cardioactive Peptides in *Drosophila melanogaster*

**DOI:** 10.3390/ijms20010002

**Published:** 2018-12-20

**Authors:** Ronja Schiemann, Kay Lammers, Maren Janz, Jana Lohmann, Achim Paululat, Heiko Meyer

**Affiliations:** Department of Zoology and Developmental Biology, University of Osnabrück, Barbarastraße 11, 49076 Osnabrück, Germany; Ronja.Schiemann@biologie.uni-osnabrueck.de (R.S.); kay.lammers@biologie.uni-osnabrueck.de (K.L.); Maren.Janz@biologie.uni-osnabrueck.de (M.J.); ja.lohmann@gmx.de (J.L.); achim.paululat@biologie.uni-osnabrueck.de (A.P.)

**Keywords:** endocrine signalling, dorsal vessel, heart function, heart physiology, neuropeptides, peptide hormones, peptide signalling

## Abstract

Neuropeptides and peptide hormones serve as critical regulators of numerous biological processes, including development, growth, reproduction, physiology, and behaviour. In mammals, peptidergic regulatory systems are complex and often involve multiple peptides that act at different levels and relay to different receptors. To improve the mechanistic understanding of such complex systems, invertebrate models in which evolutionarily conserved peptides and receptors regulate similar biological processes but in a less complex manner have emerged as highly valuable. *Drosophila melanogaster* represents a favoured model for the characterisation of novel peptidergic signalling events and for evaluating the relevance of those events *in vivo*. In the present study, we analysed a set of neuropeptides and peptide hormones for their ability to modulate cardiac function in semi-intact larval *Drosophila melanogaster*. We identified numerous peptides that significantly affected heart parameters such as heart rate, systolic and diastolic interval, rhythmicity, and contractility. Thus, peptidergic regulation of the *Drosophila* heart is not restricted to chronotropic adaptation but also includes inotropic modulation. By specifically interfering with the expression of corresponding peptides in transgenic animals, we assessed the *in vivo* relevance of the respective peptidergic regulation. Based on the functional conservation of certain peptides throughout the animal kingdom, the identified cardiomodulatory activities may be relevant not only to proper heart function in *Drosophila*, but also to corresponding processes in vertebrates, including humans.

## 1. Introduction

Peptide signalling represents an evolutionarily highly conserved mechanism for regulating numerous biological processes, including development, growth, reproduction, physiology, and behaviour [1,2,3,4,5,6]. Sessile animals with limited locomotory activities, such as Hydra, utilise distinct neuropeptides, e.g., for signal transmission between neurons and muscles [7,8,9,10]. More evolved animals, such as mammals, exhibit highly complex peptidergic signalling that is still far from being completely understood. The observations that most neuropeptides studied to date fulfil more than one function and that multiple neuropeptides must work in concert to coordinate certain physiological processes render the system extremely diverse [11]. Additionally, the recent and still ongoing identification of small open reading frames (sORFs/smORFs) present in RNAs, which were previously considered noncoding, has contributed to an increasingly complex picture, as many of these ORFs are translated into previously unknown peptides of crucial physiological functionality [12,13,14,15]. Nevertheless, in the last decades, considerable progress has been made toward understanding distinct peptidergic signalling systems [16,17,18]. In this context, invertebrate models have proven highly valuable for understanding the physiological bases of neuropeptide functionality in detail. Crustaceans and insects were commonly used to isolate biologically active peptide species and to measure their respective functionalities via specialised *in vitro* analyses [19,20,21,22,23,24,25]. More recently, the ability to specifically interfere with expression of peptide precursors or with the expression of the respective receptors has rendered *Drosophila melanogaster* a favoured organism for studying neuropeptide and peptide hormone function *in vivo* [26,27,28,29,30]. Corresponding analyses resulted in the identification of numerous mature neuropeptides that are released from individual neurohemal release sites of the *Drosophila* central nervous system [31,32,33]. In addition, a number of physiological and behavioural aspects were identified as being regulated, at least in part, by peptide signalling in *Drosophila*. These aspects include reproduction and growth [34,35,36,37,38], food intake [39,40,41], learning and memory [42,43,44], and circadian rhythmicity [45,46,47,48,49].

Despite its physiological significance, very little is known about the peptidergic regulation of the *Drosophila* heart. In former studies, we characterised the substrate specificity of Neprilysin 4 (Nep4), a heart surface bound metallopeptidase [40,50]. In the course of these investigations, we identified several peptides that were hydrolysed by Nep4 and considered cleavage by the heart bound peptidase initial indication for a putative heart modulatory function of the respective peptide. To assess the validity of this indication, in the present study, we analysed corresponding peptides for cardiomodulatory activity. Presence of the respective neuropeptides and peptide hormones in *Drosophila* has been experimentally confirmed previously [32,33,51,52,53]. As a result, we identified a number of peptides that significantly modulated heart function in semi-intact animals. Affected parameters included heart rate, systolic and diastolic interval, fractional shortening, and rhythmicity. By specifically interfering with the expression of the peptides´ precursor genes, we further assessed the *in vivo* relevance of distinct peptides. Thus, our results contribute to an in-depth understanding of the physiological mechanisms that regulate heart function in *Drosophila*. Considering the functional conservation of certain peptides and receptors, the observed cardiomodulatory activities may also be relevant to corresponding processes in vertebrates, including humans.

## 2. Results

### 2.1. The Larval Drosophila Heart Is Highly Responsive to Peptide Application

In previous studies, peptides such as Angiotensin [54], CCAP [55,56], FMRFamide-related peptides [57,58], and Proctolin [59,60,61] were identified as efficient regulators of heart function in *Drosophila*. To identify additional cardiomodulatory peptides, we analysed a set of neuropeptides and peptide hormones for their ability to modulate distinct larval heart parameters. The presence of all respective peptides in *Drosophila*, as well as their corresponding molecular masses and sequences, was experimentally confirmed in previous studies [32,33,51,52,53]. In an initial screen, we individually applied each synthesised peptide species at a concentration of 1 × 10^−7^ M onto semi-intact larval heart preparations and measured the resulting effects on characteristic heart parameters such as heart rate, systolic and diastolic interval, fractional shortening, and rhythmicity via semi-automatic optical heartbeat analysis (SOHA [62]). The tested peptides were Adipokinetic hormone (AKH); Allatostatin A1, A2, A3, and A4 (AstA1–A4); Corazonin; Diuretic hormone 31 (DH31); Drosulfakinins 1 and 2 (DSK1, DSK2); Leucokinin; Proctolin; short Neuropeptide F (sNPF1_1–11_, sNPF2_12–19_); and Tachykinin 1 to 6 (Table 1).

**Figure 1:**
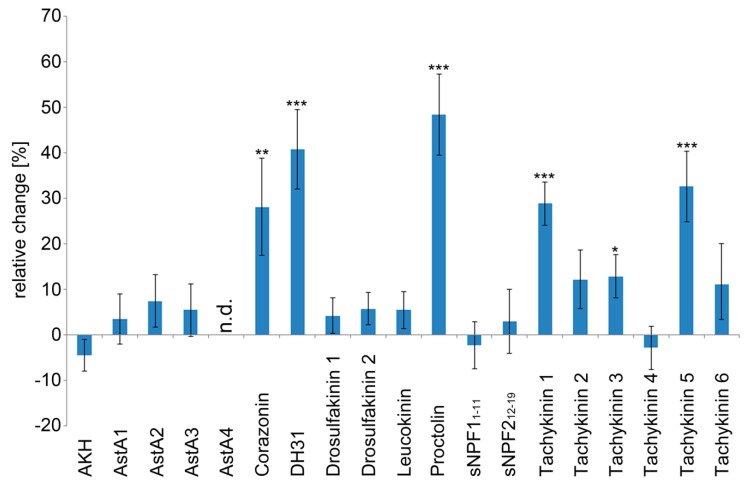
Heart rate is modulated by peptide signalling in semi-intact *Drosophila* larvae. Individual effects of the tested peptides (1 × 10^−7^ M) on heart rate. Data are presented as mean values (± S.E.M.) of the relative changes in heart rate in relation to the individual control preparations (prior to peptide application). A minimum of ten animals per peptide were measured. Significance levels are indicated by asterisks (paired sample Student´s *t*-test, * *p* < 0.05; ** *p* < 0.01; *** *p* < 0.001). Application of Allatostatin A4 resulted in a complete heartbeat arrest (heart rate not determined, n.d.).

As depicted in Figure 1, six peptides exhibited considerable cardioacceleratory activity. These were Corazonin (+28.1% heart rate increase), DH31 (+40.8% heart rate increase), Proctolin (+48.4% heart rate increase), Tachykinin 1 (+28.8% heart rate increase), Tachykinin 3 (+12.9% heart rate increase), and Tachykinin 5 (+32.6% heart rate increase). None of the tested peptides significantly reduced heart rate. Application of Allatostatin A4 resulted in a complete, yet reversible, heartbeat arrest. 

**Figure 2:**
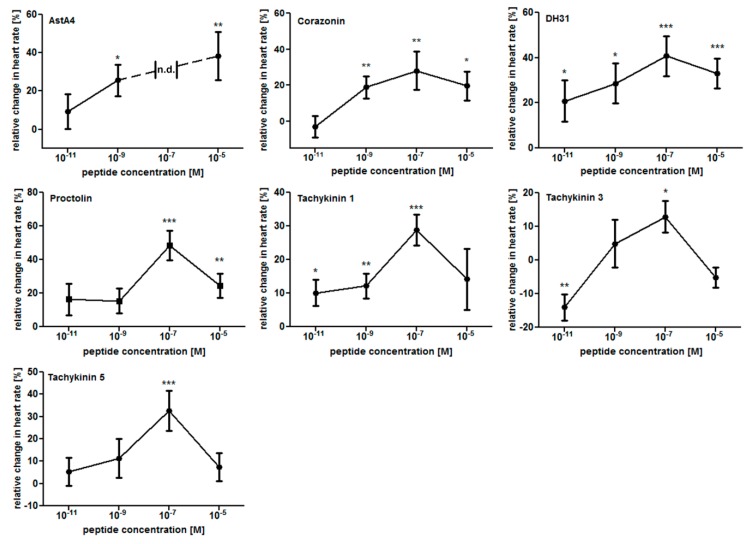
Dose–response curves for the effects of chronotropic peptides in semi-intact *Drosophila* larvae. Individual effects of the tested peptides at 1 × 10^−11^ M, 1 × 10^−9^ M, 1 × 10^−7^ M, and 1 × 10^−5^ M are shown. Data are presented as mean values (± S.E.M.) of the relative changes in heart rate in relation to the individual control preparations (prior to peptide application). A minimum of ten animals per peptide were measured. Significance levels are indicated by asterisks (paired sample Student´s *t*-test, * *p* < 0.05; ** *p* < 0.01; *** *p* < 0.001). n.d. indicates “not determined” (due to heartbeat arrest).

To further specify these results, we performed dose–response experiments with the six identified cardioacceleratory peptides as well as with Allatostatin A4. Applied peptide concentrations were 1 × 10^−11^ M, 1 × 10^−9^ M, 1 × 10^−7^ M, and 1 × 10^−5^ M. As depicted (Figure 2), most peptides progressively stimulated heart rate while their concentrations were increased from 1 × 10^−11^ M to 1 × 10^−7^ M. Only Proctolin and Tachykinin 5 required higher threshold concentrations to induce an effect (1 × 10^−7^ M). Except for AstA4, all peptides tested exhibited the strongest cardioacceleratory effect at 1 × 10^-7^ M, with a further increase in concentration resulting in a diminished response. Interestingly, AstA4 induced heartbeat arrest at this particular concentration (1 × 10^−7^ M), while 1 × 10^−9^ M and 1 × 10^−5^ M increased heart rate by 25.7% and 38.4%, respectively. In contrast to all other peptides, Tachykinin 3 decreased heart rate by 14% if applied at 1 × 10^−11^ M, while higher concentrations (1 × 10^−7^ M) elicited a cardioacceleratory response (+12.9% increased heart rate).

**Figure 3:**
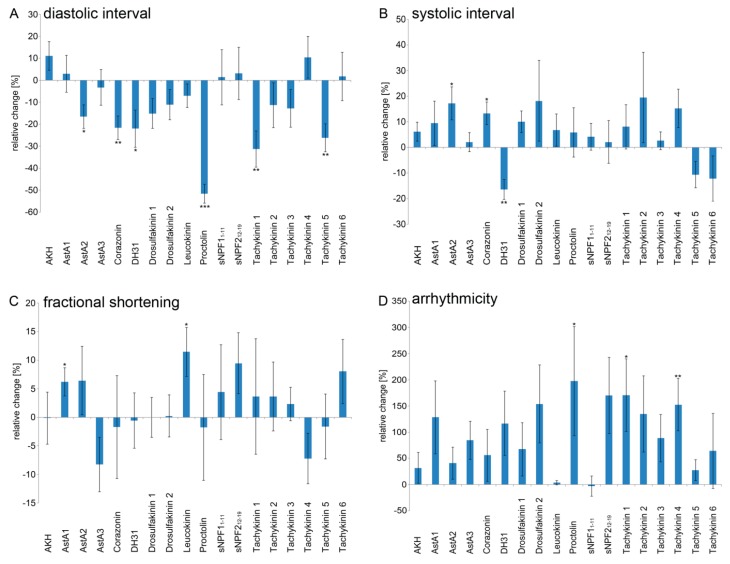
Diastolic and systolic intervals, fractional shortening and rhythmicity are modulated by peptide signalling in semi-intact *Drosophila* larvae. Individual effects of the tested peptides (1 × 10^−7^ M) on: (**A**) diastolic interval; (**B**) systolic interval; (**C**) fractional shortening; and (**D**) rhythmicity. Data are presented as mean values (± S.E.M.) of the relative changes in the respective parameters in relation to the individual control preparations (prior to peptide application). A minimum of ten animals per peptide were measured. Significance levels are indicated by asterisks (paired sample Student´s *t*-test, * *p* < 0.05; ** *p* < 0.01; *** *p* < 0.001).

To assess whether the cardioacceleratory effects were caused by a decreased diastolic or systolic interval, we also measured these parameters individually. Due to the fact that for most peptides the strongest effects on heart rate were recorded at a concentration of 1 × 10^−7^ M (Figure 2), in this set of experiments, only this concentration was applied. Among the six cardioacceleratory peptides, five significantly reduced the diastolic interval: Corazonin (−21.6%), DH31 (−22.0%), Proctolin (−51.6%), Tachykinin 1 (−31.2%), and Tachykinin 5 (−26.2%) (Figure 3A). By contrast, the systolic interval was affected only by Corazonin (elongated by 13.3%) and DH31 (shortened by 16.4%) (Figure 3B). Thus, the increased heart rates are predominantly caused by decreased diastolic intervals. Interestingly, application of Allatostatin A2 resulted in a shortened diastolic interval (−16.5%), but an elongated systolic interval (+17.2%). In combination, these opposing effects cancelled each other, resulting in an unaltered heart rate (Figure 1 and Figure 3A,B).

To assess possible effects on cardiac muscle contraction, we measured peptide-induced changes in fractional shortening. As depicted in Figure 3C, Leucokinin increased contractility by 11.4 % and Allatostatin A1 increased it by 6.2%. As a final testing parameter, we measured possible peptidergic effects on heart rhythmicity. While application of Proctolin (+197.4%), Tachykinin 1 (+170.2%), and Tachykinin 4 (+152.5%) resulted in significantly increased arrhythmia, none of the remaining peptides affected this parameter in a statistically significant manner (Figure 3D).

**Figure 4:**
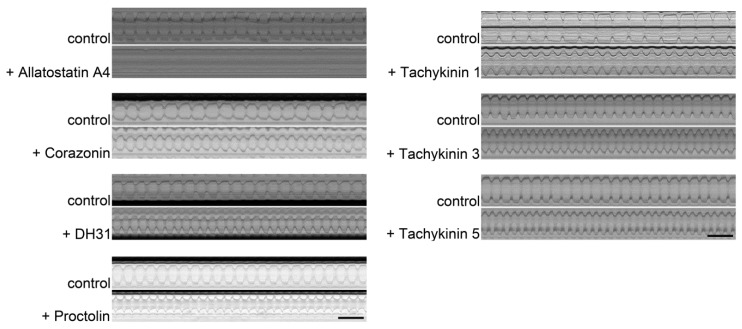
Representative M-modes of semi-intact larval *Drosophila* hearts incubated with distinct peptides (1 × 10^−7^ M). The movement of the heart walls over time (10 s) is depicted. Application of Corazonin, DH31, Proctolin, or Tachykinins 1, 3, or 5 has a cardioacceleratory effect. Application of Allatostatin A4 results in cardiac arrest. Upper panels represent control hearts (prior to peptide application); lower panels depict the same hearts 1 min after peptide application. Scale bars indicate 1 s.

The peptide-specific cardiomodulatory effects are summarised in Table 1. Corresponding raw data are presented in Table S1. Representative M-modes for the peptides that significantly affected heart rate are shown in Figure 4.

### 2.2. Peptidergic Signalling Affects Proper Heart Function In Vivo

**Figure 5:**
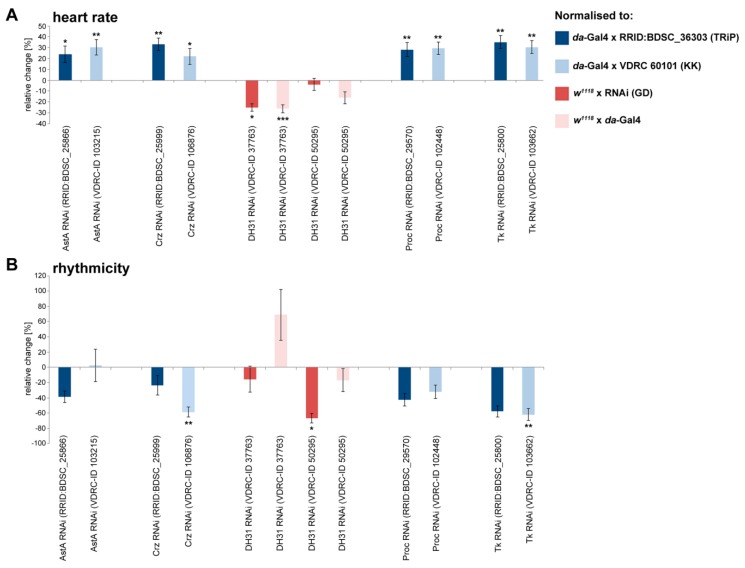
Knockdown of distinct peptide precursor proteins significantly affects heart function in intact *Drosophila* larvae. Depicted are the individual effects of peptide precursor protein knockdown on (**A**) heart rate and (**B**) rhythmicity. *daughterless*-Gal4 (*da*-Gal4) was used as a ubiquitous driver. Data are presented as mean values (± S.E.M.) of the relative changes in the respective parameters in relation to the depicted controls. At least ten animals per genotype were measured. Significance levels are indicated by asterisks (Student´s *t*-test, * *p* < 0.05; ** *p* < 0.01; *** *p* < 0.001).

As described above, 9 of the 19 peptides tested exhibited a significant modulation of chronotropic heart parameters in semi-intact animals (Allatostatin A2 and A4, Corazonin, DH31, Proctolin, and Tachykinin 1, 3, 4, and 5; Figure 1, Figure 2, Figure 3 and Figure 4 and Table 1). To analyse the physiological relevance of these effects *in vivo*, we utilised an RNAi mediated knockdown approach, with the aim of reducing abundance of the respective peptide precursor proteins. Ubiquitous *daughterless*-Gal4 was used to drive RNAi expression in any tissue possibly producing and secreting the peptide of interest. To allow for discrete evaluation of the knockdown effects *in vivo*, we analysed intact 3rd instar larvae rather than using semi-intact preparations. Of note, due to technical restrictions regarding the reproducible measurement of the heart perimeter in intact animals, which requires a direct view of the tissue without refractive epidermal cells in the optical path, the *in vivo* analysis was limited to measuring heart rate and rhythmicity. To account for possible off-target effects or variable knockdown efficiencies, we analysed at least two independent RNAi lines per gene. All lines were pre-selected for the expression of individual hairpin sequences. Significant effects were considered valid only if they resulted from two transcript-specific hairpins. As depicted in Figure 5, ubiquitous knockdown of the selected precursor proteins caused discrete effects. Considering the validity criteria depicted above, reduced production of the Allatostatin A, Corazonin, Proctolin, and Tachykinin precursor proteins resulted in a significantly increased heart rate. By contrast, expression of one hairpin specific to the *DH31* precursor transcript reduced heart rate, while the second hairpin did not elicit any significant effects (Figure 5A). Regarding heart rhythmicity, knockdown of the Corazonin, DH31, and Tachykinin precursor proteins resulted in a tendency towards a more regular heartbeat. However, in all cases, only one of the analysed hairpins caused statistically significant effects, rendering the analysis of this parameter inconclusive (Figure 5B).

To allow for a detailed evaluation of the knockdown effects, raw data for all lines tested are provided in Table S2.

## 3. Discussion

Peptidergic signalling represents an evolutionarily highly conserved means of regulating numerous fundamental biological processes. While peptidergic regulation of development, growth, reproduction, and behaviour has been investigated extensively, few studies to date have looked at peptide-based modulation of cardiac function. However, peptide-mediated effects on heart rate and heart contraction have repetitively been identified in *Drosophila* [54,57,58,59,60,61], with the corresponding peptides acting via distinct G protein-coupled receptors (GPCRs) that in turn activate specific cellular signal transduction pathways [63]. In addition, several studies report on peptide-mediated regulation of cardiac rhythmicity in humans: Endothelin-1 or Angiotensin II are well-known for their ability to modulate heart rhythmicity [64,65,66,67,68,69]. Furthermore, natriuretic peptides affect cardiac rhythmicity, most likely by regulating SERCA activity via cGMP and PKG mediated phosphorylation of Phospholamban. Impairments in this regulation result in cardiac arrhythmia [70,71]. In the present study, we screened a number of peptides evidentially produced in *Drosophila melanogaster* for their ability to modulate corresponding heart parameters in 3rd instar larvae. We selected this stage of animal development due to the myogenic nature of the larval heart [72]. Thus, all effects described herein should be either intrinsic to heart or based on endocrine signalling, and do not involve any direct innervation component. Among the 19 peptides tested, 11 significantly modulated larval heart function in semi-intact preparations (Figure 1, Figure 2 and Figure 3 and Table 1). While at first sight this ratio of cardioactive peptides appears to be rather high, it is likely consequential of the fact that we pre-selected the tested peptides and analysed only factors that have previously been identified as substrates of the heart surface bound peptidase Nep4 [40]. Since we consider cleavage by the peptidase indicative of a heart modulatory function, the high proportion of cardioactive peptides becomes more allegeable. In this respect, our results that, except for Tachykinin 3, all identified peptides acted exclusively as positive regulators of chronotropic (Figure 1 and Figure 2) or inotropic heart parameters (Figure 3C) may suggest that the peptidergic regulation of larval heart physiology is largely restricted to augmenting respective parameters, while reduced heart rate/heart contractility is predominantly achieved through the removal of corresponding peptides from the haemolymph. The latter likely involves hydrolysis by certain peptidases present at the surface of muscle or heart cells, such as Nep4 [40,50]. Of note, some of the identified peptides affected multiple cardiac parameters, while others exhibited more functional specificity. For example, Proctolin and Tachykinin 1 modified heart rate and rhythmicity, while Corazonin, DH31, and Tachykinin 5 specifically increased heart rate. By contrast, Tachykinin 4 affected only heart rhythmicity (Figure 1, Figure 2 and Figure 3 and Table 1). Interestingly, the only two peptides that altered fractional shortening, Allatostatin A1 and Leucokinin (Figure 3C), also exclusively modulated this parameter. Considering the fact that vice versa none of the chronotropic peptides affected fractional shortening (Table 1), these observations suggest that activity of cardiomodulatory peptides in general is either specific to chronotropic or to inotropic modulation. In addition, these results indicate a complex peptidergic regulation of the larval *Drosophila* heart that involves chronotropic and inotropic adaptation and requires simultaneous signalling from several specific factors. With regard to inotropic modulation, we identified Allatostatin A1 and Leucokinin as regulatory peptides. Interestingly, leucokinins have already been associated with muscle contractility and a stimulatory effect of the peptides on hindgut contraction in *Leucophaea maderae* has been reported [73,74]. The maximum response for each leucokinin species was recorded at 2.1 × 10^−7^ M; however, the heart did not respond to any of the peptides. By contrast, our data clearly show that Leucokinin increases the contractility of the larval *Drosophila* heart at comparable concentrations (1 × 10^−7^ M, Figure 3C). Thus, while the general effect on muscle tissue seems to be conserved, leucokinins apparently exhibit species-dependent tissue specificities, probably based on the presence or absence of a corresponding receptor at the surface of a given tissue.

Regarding *Drosophila* tachykinins (DTKs), two receptors have been identified: NKD (neurokinin receptor from *Drosophila*), and DTKR (*Drosophila* tachykinin receptor). With respect to NKD, it has been shown that it can be activated only by DTK-6 [75]. Since we found that DTK-6 application did not affect cardiac activity (Figure 1 and Figure 3), participation of NKD in the peptidergic regulation of the *Drosophila* heart appears unlikely. By contrast, DTKR has been reported to be responsive to all six *Drosophila* tachykinins, with DTK-1 inducing the strongest response (EC_50_ = 3.9 × 10^−9^ M) and DTKs -2 to -6 being about one order of magnitude less active [76]. This broader specificity along with our results that numerous *Drosophila* tachykinins affected heart function (DTK-1, -3, -4, and -5; Figure 1, Figure 2 and Figure 3) indicates that cardiac-specific tachykinin signalling is mainly relayed via DTKR. Interestingly, DTK-2 and DTK-6 did not elicit any significant effect, although they have been reported to activate DTKR expressed in HEK-293 cells [76]. This result indicates that DTK-2 and -6 may have additional functions in *Drosophila* larvae, probably mediated by binding to a receptor different from NKD or DTKR, which attenuate the effects of DTKR activation in cardiac tissue. Concentration dependent activation of distinct receptors may also be causal to our observation that DTK-3 reduces heart rate at 1 × 10^−11^ M, but increases the same parameter at 1 × 10^−7^ M (Figure 2). With respect to allatostatins, a similar situation may be present. Also for these peptides, two receptor proteins have been identified in *Drosophila* (DAR-1 and DAR-2). According to Ca^2+^ mobilisation assays in transfected CHO cells, both receptors exhibit EC_50_ values in the range of 20–100 nM for all allatostatins tested (AstA1–AstA4 [77]). Thus, similar cardiomodulatory effects would be expected for these peptides if DAR-1 and DAR-2 were the only responsive receptors in *Drosophila*. Interestingly, we observed a significant effect on heart function only in response to AstA2 and AstA4 application, while AstA1 and AstA3 did not affect any chronotropic heart parameter (Figure 1 and Figure 3). This lack of activity observed for two of the four tested allatostatins indicates that neither DAR-1 nor DAR-2 is involved in regulating larval *Drosophila* heartbeat. Consequently, the distinct effects of AstA2 and AstA4 application are presumably mediated by activation of yet unknown receptors. As confirmed by dose–response experiments, AstA4 mediated activation of such a putative receptor resulted in an increased heart rate at ligand concentrations of 1 × 10^−9^ M and 1 × 10^−5^ M (Figure 2). Interestingly, at 1 × 10^−7^ M a complete, yet reversible heartbeat arrest occurred, indicating an unphysiologically strong response at this particular AstA4 concentration that may result in stress-induced heart failure.

To assess the *in vivo* relevance of these data in more detail, we analysed the physiological effects of all chronotropic peptides identified in our screen (Allatostatin A2 and A4, Corazonin, DH31, Proctolin, and Tachykinin 1, 3, 4, and 5) in intact transgenic larvae. In this respect, we used ubiquitous RNAi (*daughterless*-Gal4) to knockdown expression of the respective precursor proteins in any tissue possibly producing and secreting the peptide of interest. As shown in Figure 5, knockdown of all peptide precursors affected heart rate, which substantiates a cardiomodulatory activity of the mature peptides. Interestingly, only DH31 precursor knockdown resulted in a tendency to decrease this parameter, while reduced levels of the Allatostatin, Corazonin, Proctolin, and Tachykinin precursor increased heart rate (Figure 5A). These results are surprising, since our data from semi-intact heart preparations indicate a cardioacceleratory activity of all respective peptides (Figure 1 and Figure 2). Thus, vice versa, knockdown of the respective precursor proteins should result in decreased heart rate. However, given the fact that neuropeptides typically have different roles during development and adulthood, the observed knockdown effects may reflect a combination of physiological and developmental impairments. In addition, knockdown of a precursor protein usually affects numerous mature peptides. This simultaneous loss of peptide signalling, probably interfering with multiple peptidergic systems, may cause considerable physiological stress that is sensed by the heart and results in an increased heart rate. On the other hand, the type of assay may also influence the effects on heart rate. In *Cancer magister*, it has been shown that different experimental setups can affect the results, as Proctolin application in intact animals decreased heart rate, while semi-isolated hearts exhibited a cardioacceleratory response [78]. At least with respect to Proctolin, this discrepancy is observed also in *Drosophila*: while Proctolin injection into 3rd instar larvae is reported to decrease heart rate [61], our results using semi-intact animals clearly show a cardioacceleratory activity of the peptide (Figure 1 and Figure 2). Thus, a combination of methodical setups appears to be required to adequately characterise cardioactive peptides in *Drosophila*. However, considering the reduced complexity and the resulting gain in interpretability, we regard direct application analyses using semi-intact heart preparations as favourable techniques for initial peptide identification. 

By combining semi-intact heart preparations with *in vivo* analyses, we have identified a number of cardioactive peptides in *Drosophila melanogaster*. While in-depth analyses of the distinct physiological functions of these peptides require further efforts, our data represent a valuable resource for designing corresponding studies in the future.

## 4. Materials and Methods

### 4.1. Fly Strains

*daughterless*-Gal4 (*da*-Gal4, RRID:BDSC_55850) was used as a ubiquitous driver. RNAi lines (Table 2) were obtained from either the Vienna *Drosophila* Resource Center (VDRC) or the Bloomington *Drosophila* Stock Center (BDSC). The KK collection specific host strain VDRC-ID 60101 was used as a control for the KK RNAi lines, and the strain BDSC_36303 served as a control for the TRiP RNAi lines (each of them crossed to *da*-Gal4). Both control lines share identical genetic backgrounds with the lines of the respective collection (KK or TRiP). The genetically distinct lines of the GD collection (Table 2) were controlled by crossing both, the respective UAS-RNAi lines as well as the applied driver line (*da*-Gal4) to *w^1118^* (RRID:BDSC_5905). 

### 4.2. Peptide Application Assay and Dose–Response Analysis

Prior to peptide application, wandering male 3rd instar larvae were dissected in artificial haemolymph (5 mM KCl, 8 mM MgCl_2_, 2 mM CaCl_2_, 108 mM NaCl, 1 mM NaH_2_PO_4_, 5 mM HEPES and 4 mM NaHCO_3_, pH 7.1). Prior to use, the buffer was supplemented with trehalose (final concentration: 5 mM) and sucrose (final concentration: 10 mM) [79]. Specimens were pinned down with the ventral side upwards onto Sylgard 184 silicone elastomer plates. All internal organs except for the heart and associated tissue (e.g. alary muscles, pericardial cells) were removed. After a resting period of 10 min, a 60 s video of the heartbeat was recorded and used as a control. Afterwards, the artificial haemolymph was removed and replaced with artificial haemolymph containing a candidate peptide (Table 1). After 1 min, the heartbeat was again recorded for 60 s and analysed for peptide-specific alterations in comparison to the respective control measurement. For an initial screen, only one peptide concentration was tested (1 × 10^−7^ M). To further assess the dose–responsive relationship of positive candidates, three additional concentrations (1 × 10^−11^ M, 1 × 10^−9^ M and 1 × 10^−5^ M) of the respective peptides were applied. All peptides were synthesised by JPT Peptide Technologies (Berlin, Germany) and were of >90 % purity. The proper sequence and mass of each peptide was confirmed using an ESI-ion trap (Amazon ETD Speed with a captive spray ionisation unit, Bruker Corporation, Billerica, MA, USA) by measuring the masses of the intact molecules as well as the masses of the fragments, which were generated by collision-induced dissociation (CID) of the corresponding parent ion.

### 4.3. In Vivo Measurement of Heart Parameters

To measure the heart function of animals with ubiquitous knockdown of the peptide precursors or cardiac-specific knockdown of their receptors *in vivo*, wandering male 3rd instar larvae were anaesthetised for 8 min using ether. The anaesthetised animals were transferred with the dorsal side upwards to a moistened microscope slide and a 60 s video of the beating heart was captured through the cuticle. For optimal detection of the heartbeat, the camera was focused on a region in which the heart tube was in close proximity to trachea (dorsal trunks) or fat body tissue, allowing the simultaneous detection of the heartbeat and related movements of the surrounding tissues with high contrast.

### 4.4. Video Analysis and Calculation of Cardiac Parameters

For heartbeat recording, a high-speed video camera (Basler piA-640) was mounted onto an upright microscope (Leica DMLB) equipped with a 10× Leica Fluotar. Movies were captured with the software Firecapture ® (freeware by Torsten Edelmann) and processed with ImageJ [80]. 

For the peptide screen, heart parameters were analysed using SOHA (semi-automated optical heartbeat analysis), a MATLAB application introduced by Fink et al. [62] and further developed by Ocorr et al. [81]. The SOHA software utilises two computer algorithms to combine overall darkness changes of a video (Frame Brightness Algorithm) with pixel-by-pixel intensity changes (Changing Pixel Intensity Algorithm), thereby allowing calculation of different heart beat parameters. Besides the heart rate [Hz] and systolic/diastolic intervals (duration of contraction and relaxation phases [ms]), also contractility ([%] of fractional shortening) as well as heart beat arrhythmia can be determined. To measure fractional shortening, the SOHA software allows manually annotating the edges of the heart tube at its maximally dilated and maximally contracted state. Based on these diameters, percentages of fractional shortening can be calculated:

(1) $$\%\mathrm{FS}=\frac{\mathrm{Diastolic}\;\mathrm{diameter}-\mathrm{Systolic}\;\mathrm{diameter}}{\mathrm{Diastolic}\;\mathrm{diameter}}\;\times 100$$

As described in [62], the arrhythmicity index was calculated via dividing the standard deviation of the heart period by the median of the heart period:

(2) $$\mathrm{AI}=\frac{\mathrm{Standard}\;\mathrm{deviation}\;\left(\mathrm{HP}\right)}{\mathrm{Median}\;\left(\mathrm{HP}\right)}$$

Additionally, the SOHA software also generates kymographs (M-modes) of heart wall movements (X-axis) over time (Y-axis). M-modes are horizontally aligned pixel slices from each frame of a respective heart beat video. In this way, an M-mode allows visualising distinct differences in heart performance (e.g. heart rate). Additional data analysis was done using Microsoft Excel and GraphPad Prism 5 (GraphPad Software, La Jolla, CA, USA).

Analysis of in vivo heart parameters was done by measuring the changes in light intensity that occur during a single heartbeat. For automated evaluation of the parameter values, a self-compiled Java script with OpenCV (Open Source Computer Vision, [82]) was employed. The resulting plots (Figure S1) were verified for proper heartbeat detection. Additional data analysis was done using Microsoft Excel.

### 4.5. Statistics

Statistical analysis was performed using Microsoft Excel. For the peptide screen, a minimum of 10 animals per peptide were measured. Paired sample t-tests were performed to determine the statistical significance of peptide-specific effects. For statistical analysis of in vivo effects, an independent samples t-test was used. At least 10 animals per genotype were analysed.

## Figures and Tables

**Table 1:** Peptides used in this study and corresponding effects on distinct heart parameters in semi-intact larvae.

Peptide	Sequence	Heart Rate	Diastolic Interval	Systolic Interval	Fractional Shortening	Arrhythmicity Index
AKH	QLTFSPDWa	*/*	*/*	*/*	*/*	*/*
AstA1	VERYAFGLa	*/*	*/*	*/*	+	*/*
AstA2	LPVYNFGLa	*/*	-	+	*/*	*/*
AstA3	SRPYSFGLa	*/*	*/*	*/*	*/*	*/*
AstA4	TTRPQPFNFGLa	Θ	Θ	Θ	Θ	Θ
Corazonin	QTFQYSRGWTNa	++	--	+	*/*	*/*
DH31	TVDFGLARGYSGTQEAKHRMGLAAANFAGGPa	+++	-	--	*/*	*/*
DSK1	FDDYGHMRFa	*/*	*/*	*/*	*/*	*/*
DSK2	GGDDQFDDYGHMRFa	*/*	*/*	*/*	*/*	*/*
Leucokinin	NSVVLGKKQRFHSWGa	*/*	*/*	*/*	+	*/*
Proctolin	RYLPT	+++	---	*/*	*/*	+
sNPF1_1-11_	AQRSPSLRLRFa	*/*	*/*	*/*	*/*	*/*
sNPF1_4-11_ sNPF2_12-19_	SPSLRLRFa	*/*	*/*	*/*	*/*	*/*
Tachykinin 1	APTSSFIGMRa	+++	--	*/*	*/*	+
Tachykinin 2	APLAFVGLRa	*/*	*/*	*/*	*/*	*/*
Tachykinin 3	APTGFTGMRa	+	*/*	*/*	*/*	*/*
Tachykinin 4	APVNSFVGMRa	*/*	*/*	*/*	*/*	++
Tachykinin 5	APNGFLGMRa	+++	--	*/*	*/*	*/*
Tachykinin 6	AALSDSYDLRGKQQRFADFNSKFVAVRa	*/*	*/*	*/*	*/*	*/*

Listed are all peptides applied in this study along with their respective sequence and the effects on individual heart parameters (at 1 × 10^−7^ M). Nomenclature indicates significance of the observed effects (**+** = increase, *p* < 0.05; **++** = increase, *p* < 0.01; **+++** = increase, *p* < 0.001; **-** = decrease, *p* < 0.05; **--** = decrease, *p* < 0.01; **---** = decrease, *p* < 0.001; ***/*** = no significant effect; paired sample Student´s *t*-test). **Θ** indicates full arrest of heartbeat upon peptide application.

**Table 2:** RNAi lines used in this study.

Provided by	Collection	Identifier	Target Gene
VDRC	KK	VDRC-ID 103215	CG13633; Allatostatin A
VDRC	GD	VDRC-ID 50295	CG13094; Diuretic hormone 31
VDRC	GD	VDRC-ID 37763	CG13094; Diuretic hormone 31
VDRC	KK	VDRC-ID 106876	CG3302; Corazonin
VDRC	KK	VDRC-ID 102488	CG7105; Proctolin
VDRC	KK	VDRC-ID 103662	CG14734; Tachykinin
BDSC	TRiP	RRID:BDSC_25800	CG14734; Tachykinin
BDSC	TRiP	RRID:BDSC_25866	CG13633; Allatostatin A
BDSC	TRiP	RRID:BDSC_25999	CG3302; Corazonin
BDSC	TRiP	RRID:BDSC_29570	CG7105; Proctolin

## Supplementary Materials

The following are available online at http://www.mdpi.com/1422-0067/20/1/2/s1, Figure S1: Representative plots of heartbeat detection in intact animals: (A) *da*-Gal4 x BDSC_60101 (control, KK); and (B) *da*-Gal4 x BDSC_103215 (KK). Detected heartbeats (black dots) are labelled by consecutive numbers. In contrast to control animals (A), knockdown of the Allatostatin precursor protein results in increased heart rate (B); Table S1: Raw data of peptide-specific cardiomodulatory effects in semi-intact animals; Table S2: Raw data of knockdown-specific cardiomodulatory effects in intact animals.
