# Supplementary figures and images for: Identification and In Vivo Characterisation of Cardioactive Peptides in Drosophila melanogaster
Source: Int J Mol Sci. 2018 Dec 20;20(1):2. doi: 10.3390/ijms20010002 (PMC6337577; doi:10.3390/ijms20010002)

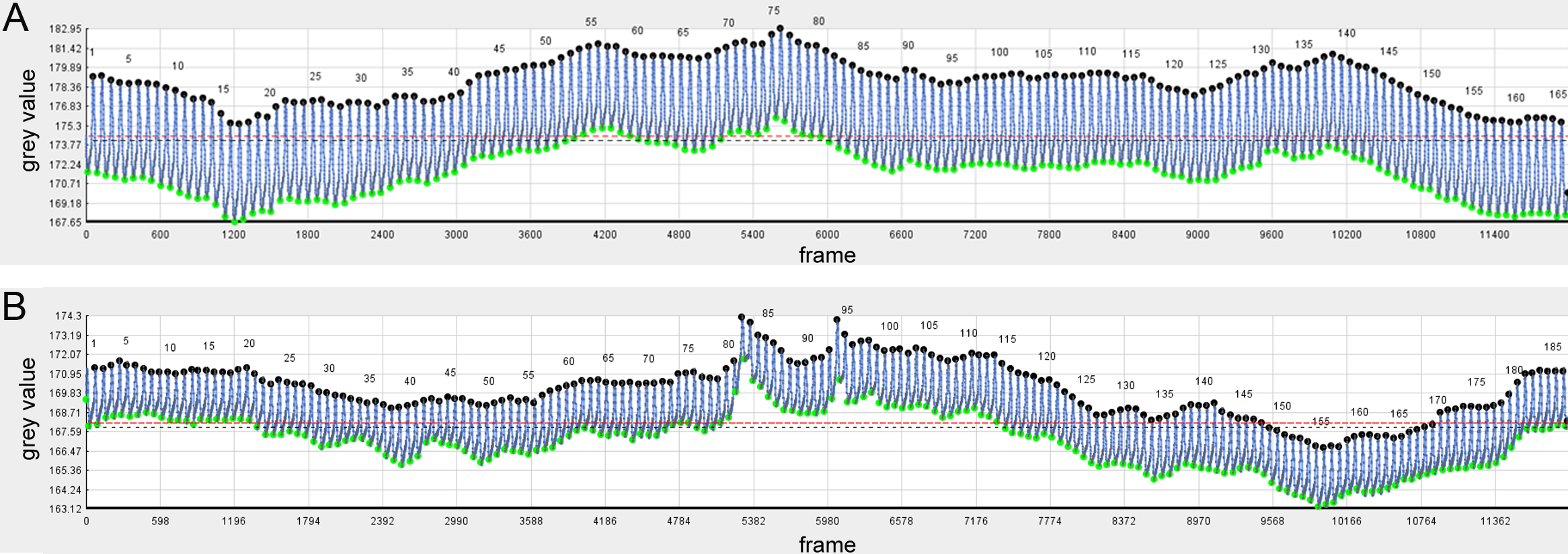

Supplement: Supplementary file 1 [file ijms-20-00002-s001.zip › Figure S1.tif]
